# Comparison between the modelled response of primary motor cortex neurons to pulse-width modulated and conventional TMS stimuli*

**DOI:** 10.1109/EMBC46164.2021.9629605

**Published:** 2021-11-01

**Authors:** Karen Wendt, Majid Memarian Sorkhabi, Jacinta O’Shea, Hayriye Cagnan, Timothy Denison

**Affiliations:** MRC Brain Network Dynamics Unit, University of Oxford, Oxford, OX1 3TH, UK; Wellcome Centre for Integrative Neuroimaging (WIN), Oxford Centre for Human Brain Activity (OHBA), University of Oxford Department of Psychiatry, Warneford Hospital, Warneford Lane, Oxford, UK; MRC Brain Network Dynamics Unit, University of Oxford, Oxford, OX1 3TH, UK

## Abstract

In this study, the neural response to pulse-width modulated (PWM) transcranial magnetic stimulation (TMS) is estimated using a computational neural model which simulates the response of cortical neurons to TMS. The recently introduced programmable TMS uses PWM to approximate conventional resonance-based TMS pulses by fast switching between voltage levels. The effect of such stimulation on the six cortical layers is modelled by estimating the activation threshold of the neurons. Modelling results are compared between the novel device and that of conventional TMS stimuli generated by Magstim stimulators. The neural responses to the PWM pulses and the conventional stimuli show a high correlation, which validates the use of pulse-width modulated pulses in TMS.

**Clinical Relevance:**

This computational modelling study demonstrates an equivalent effect of PWM and conventional TMS pulses on the nervous system which paves the way to more flexibility in exploring and choosing stimulation parameters for TMS treatment.

## I Introduction

Transcranial magnetic stimulation (TMS) is a non-invasive technique that uses the fundamental principles of magnetic induction to modulate the nervous system. Intuitively, a TMS coil can be viewed as a transformer: applying a voltage to the coil causes a current to flow through it, generating a changing magnetic field which induces a voltage in the brain tissue underneath the coil [1]. TMS is a useful tool to study the brain and to treat various psychiatric and neurological disorders [2].

Conventional TMS devices usually generate damped cosine pulses defined by the resonance period of the circuit, which limits the possible pulse shapes and patterns. Expanding the stimulation parameter space may increase the research and treatment capabilities of TMS. Our group has developed a novel TMS device called the programmable TMS (pTMS), which employs pulse-width modulation (PWM) to rapidly switch between voltage levels, generating magnetic pulses of arbitrary shapes [3]. The circuit includes an H-bridge inverter which can generate stimulus waveforms using three voltage levels. A second-generation device using cascaded H-bridges can generate five different voltage levels and deliver higher maximum energy. Using this architecture, a reference waveform of any arbitrary shape can be approximated using pulse-width modulation. The rectangular voltage pulses produced by PWM have a high-frequency content which resonance-based stimuli do not have. The low-pass filtering properties of the nervous system are expected to filter out the high frequency harmonics, resulting in an equivalent effect of the PWM pulses on neurons as compared to conventional TMS pulses.

In a recent computational model [4] a realistic finite element method model of a human head is combined with morphologically-realistic models of cortical neurons to quantify the neural response to TMS [5]. This model allows to estimate the threshold of activation of cortical neurons in response to different TMS pulses. In this study, we use this computational model to compare the neural response to magnetic stimuli generated using PWM and conventional magnetic stimulators.

## II Methods

### A Computational model

The computational model used for this study is available on Github [4] and has been described in detail in [5]. The model is based on the assumption that the quasi-static approximation may be used to calculate potentials generated by neural stimulation [6]. Thus, the electric field induced by TMS can be separated into its spatial and temporal components.

For the neurons, multi-compartment models of the neuron types found in the cortical layers 1-6 were adapted from the Blue Brain Project to match the properties of human neurons, and five clones of each cell type were created by varying their morphologies stochastically to represent the diversity within each cell type [7]. A region of interest around the motor hand knob in the primary motor cortex was populated with 3000 neurons of each type. For this, the cell body of each neuron clone was centered within a surface element of its respective layer, aligning its somatodendritic axis with the element normal, and rotated randomly around its somatodendritic axis. Additional rotations could be included for each neuron model as demonstrated in [5], however to reduce the computational time, only one rotation was included here.

**Fig. 1:**
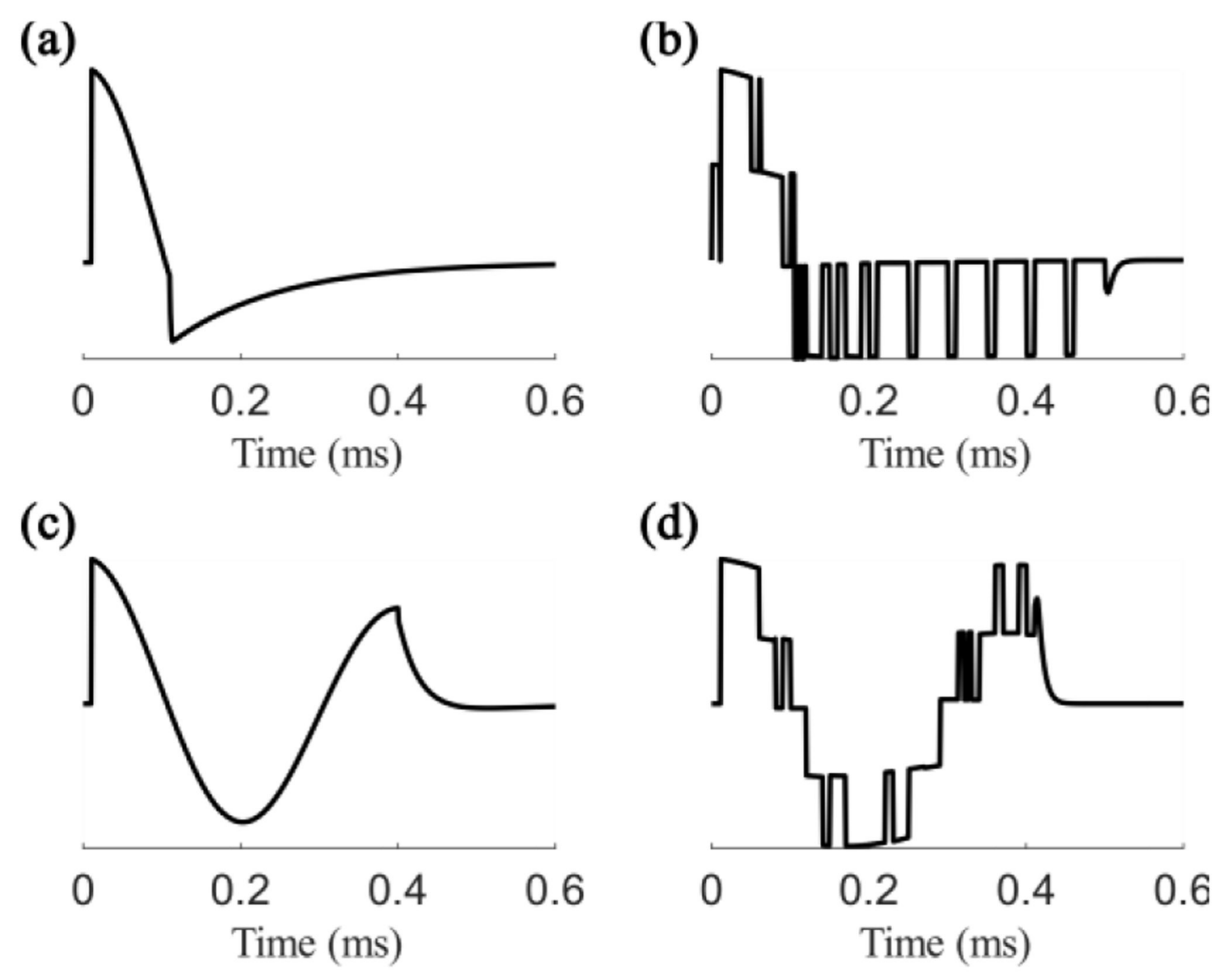
Simulated waveforms from (a) the monophasic Magstim 200 and (b) its pulse-width modulated equivalent, (c) the biphasic Magstim Rapid^2^ and (d) its pulse-width modulated equivalent using the five-level pTMS architecture.

Fig. 1 gives a schematic overview of how the model was used in this work. In brief, the spatial component of the electric field was computed (see Methods section B.) and used to calculate quasipotentials at the compartment centers of each neuron within the region of interest. These were then applied to the neuron compartments as extracellular potentials in the NEURON simulation environment [8]. The spatial distribution was scaled over time by the temporal component of the electric field according to the simulated waveform. The membrane potentials were calculated with the backward Euler method (time step: dt = 5 µs) after equilibrating to steady state. A neuron was considered activated if the membrane potential of at least three of its compartments crossed 0 mV with a positive slope. To find the activation threshold of a neuron, a binary search algorithm was used to scale the coil current’s rate of change at the pulse onset to find the minimum intensity needed to activate the neuron. The reader is referred to [5] for more details on the computational model.

### B Spatial component of electric field

The spatial component of the electric field was calculated on the example data set of a healthy subject in SimNIBS [9] as in [5]. To compare the different stimulus waveforms in this study, the electric field distribution of the Magstim 70 mm figure-of-8 coil (Magstim Company Ltd, UK), when positioned over the hand knob representation of the left primary motor cortex and oriented at 45º to the midline, was used for all simulations. The coil-to-scalp distance was set to 2 mm and the coil current’s rate of change to 1 A/μs. The primary induced current direction was set to posterior-anterior (PA) for monophasic waveforms and to anterior-posterior (AP) for biphasic waveforms according to experimental data showing lower motor thresholds in the hand muscle for these directions [5, 10].

### C Temporal component of electric field

**Fig. 2:**
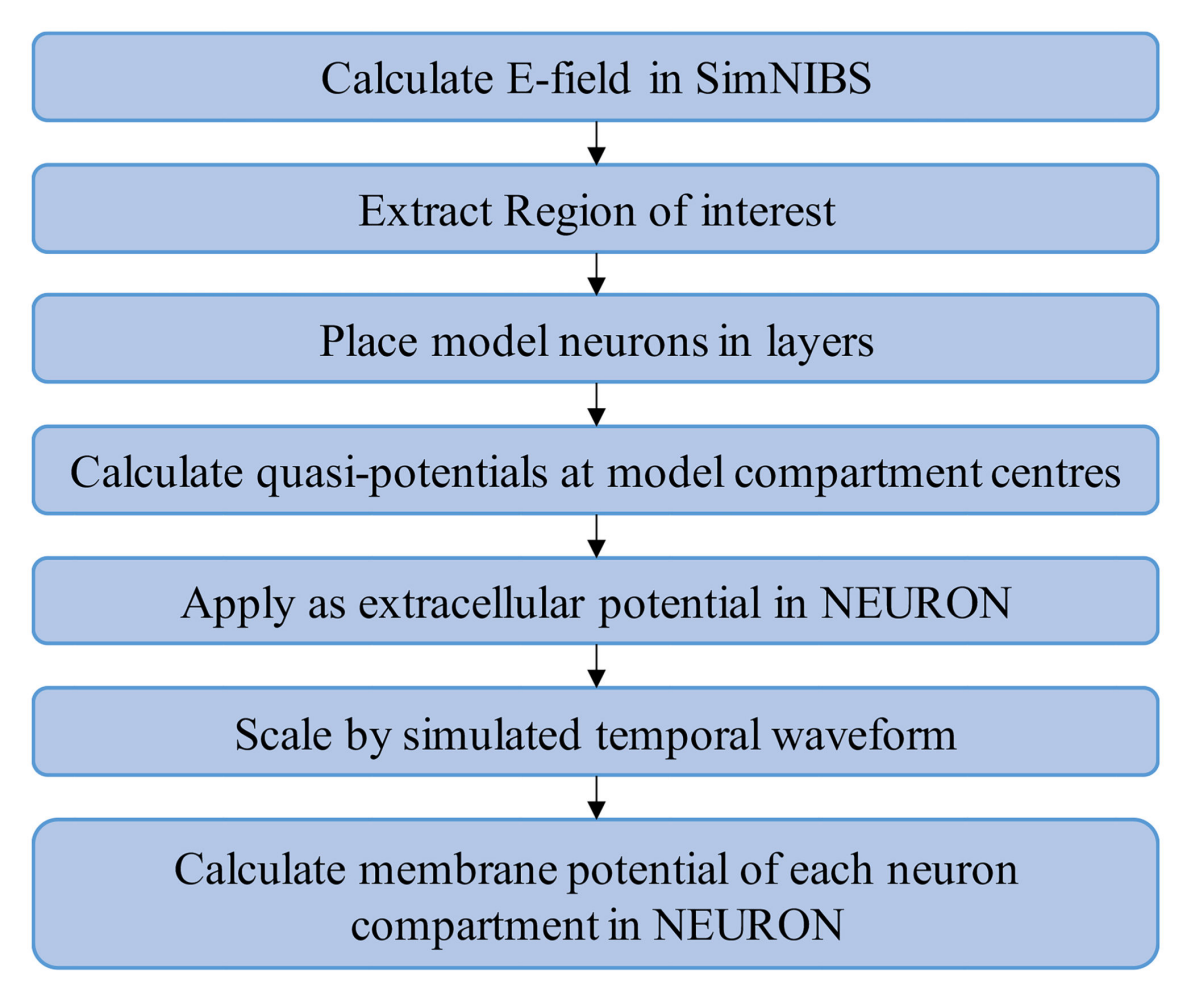
Flowchart summarizing the workflow of the computational model [3] as used on this study.

The temporal component of the electric field was simulated in Simulink in MATLAB (R2019a & R2020a, The Mathworks, Inc., Natick, MA, USA) with 1 μs time steps. The circuits of two commonly used stimulators, the Magstim 200 and the Magstim Rapid^2^ (Magstim Company Ltd, UK), and their PWM equivalents using the 5-level pTMS architecture, were modelled using the Powergui block set in Simulink. The resulting electric field waveforms (Fig. 2) were down-sampled with 5 μs time steps and the amplitude normalized before being inserted into the model as described above.

### D Data Analysis

The data analysis in this paper was conducted using the functions available in the Github repository of the computational model [4]. This includes an estimation of the cortical region that represents the first dorsal interosseous muscle in the right hand and the visualization of a 2D cross section of the crown of the pre-central gyrus. Using this, the median thresholds for each waveform and current direction are compared across each cortical layer.

Additionally, linear regression was used to quantify the relationship between the activation thresholds for each device for the same waveform type and current direction.

## III Results and Discussion

This study directly compares the modelled effects of TMS on cortical neurons using PWM versus conventional TMS using damped cosine pulses. All parameters in the model, including the spatial distribution of the electric field are kept constant, while the temporal component of the electric field is varied according to the specific device, pulse waveform and current direction.

**Fig. 3:**
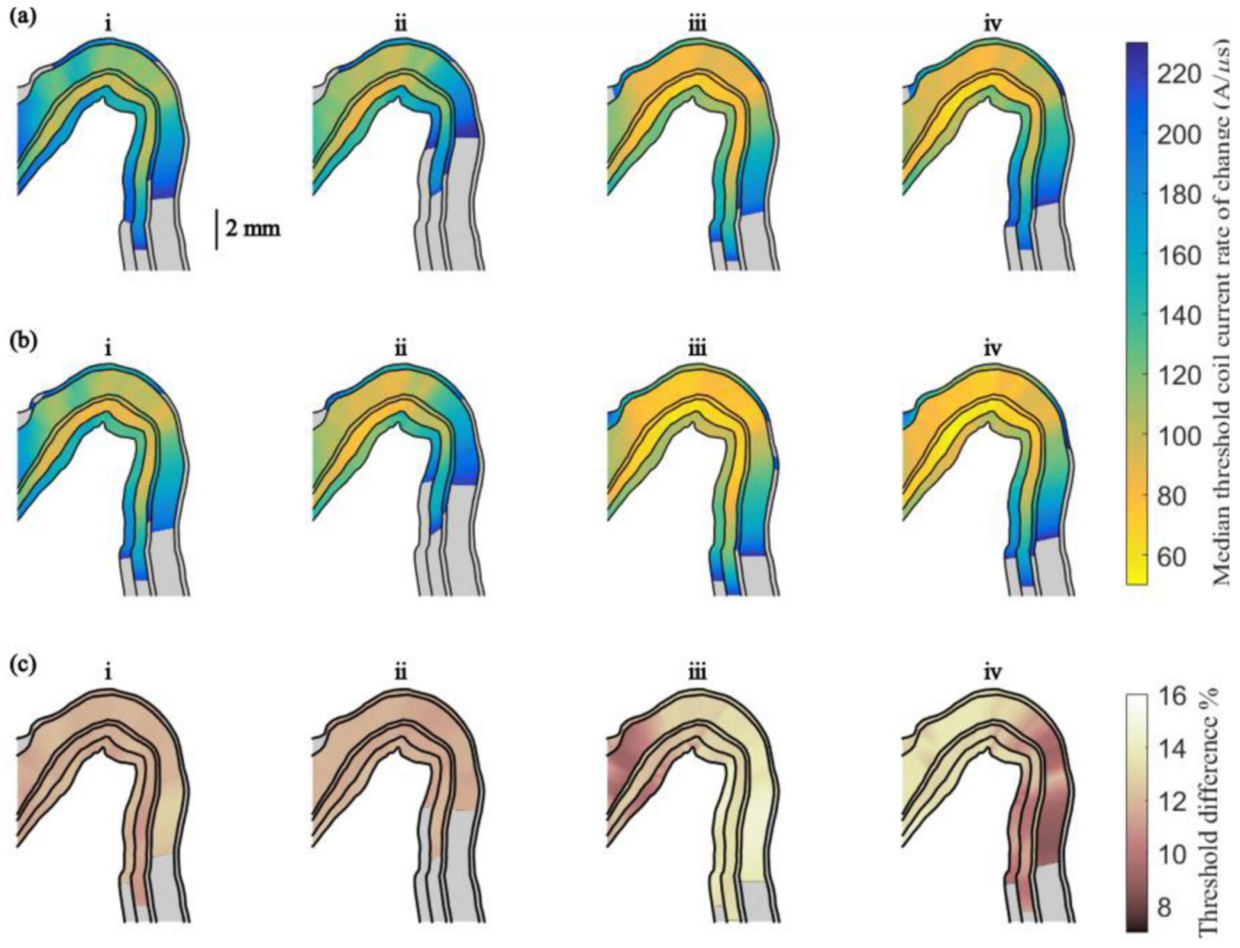
The median thresholds of the change in coil current for the six cortical layers are shown on a 2D cross section of the crown of the pre-central gyrus on a plane parallel to the stimulation coil orientation. **(a)** shows the thresholds for the pulse waveforms from the Magstim devices for **i**. monophasic stimulation in the PA direction, **ii**. monophasic stimulation in the AP direction, **iii**. biphasic stimulation in the AP direction and **iv**. biphasic stimulation in the PA direction. **(b)** shows the thresholds for 5- level pulse-width modulated approximations of each of the pulses in **(a)**. The thresholds are given in A/μs and thresholds above 230 A/μs are displayed in grey. **(c)** shows the percent difference in median thresholds between the conventional and pulse-width modulated pulses.

Fig. 3 (a) i-iv displays the median excitation thresholds for waveforms generated by conventional Magstim stimulators across the 2D cross section of the pre-central crown for monophasic PA and AP stimulation and biphasic AP and PA stimulation, respectively. Fig. 3 (b) i-iv shows the median thresholds for the corresponding pulse-width modulated pulses generated by the pTMS architecture. The thresholds from the two devices differ from 7.6-87 A/μs, which corresponds to 8.6-14.6% for different neurons (Fig. 3 (c)), with the thresholds for the monophasic stimuli differing uniformly across all neurons in the different layers (standard deviation below 0.5%). This suggests that decreasing the stimulation intensity of the programmable TMS device by around 11% should achieve an equivalent neural response to the Magstim 200.

**Fig. 4:**
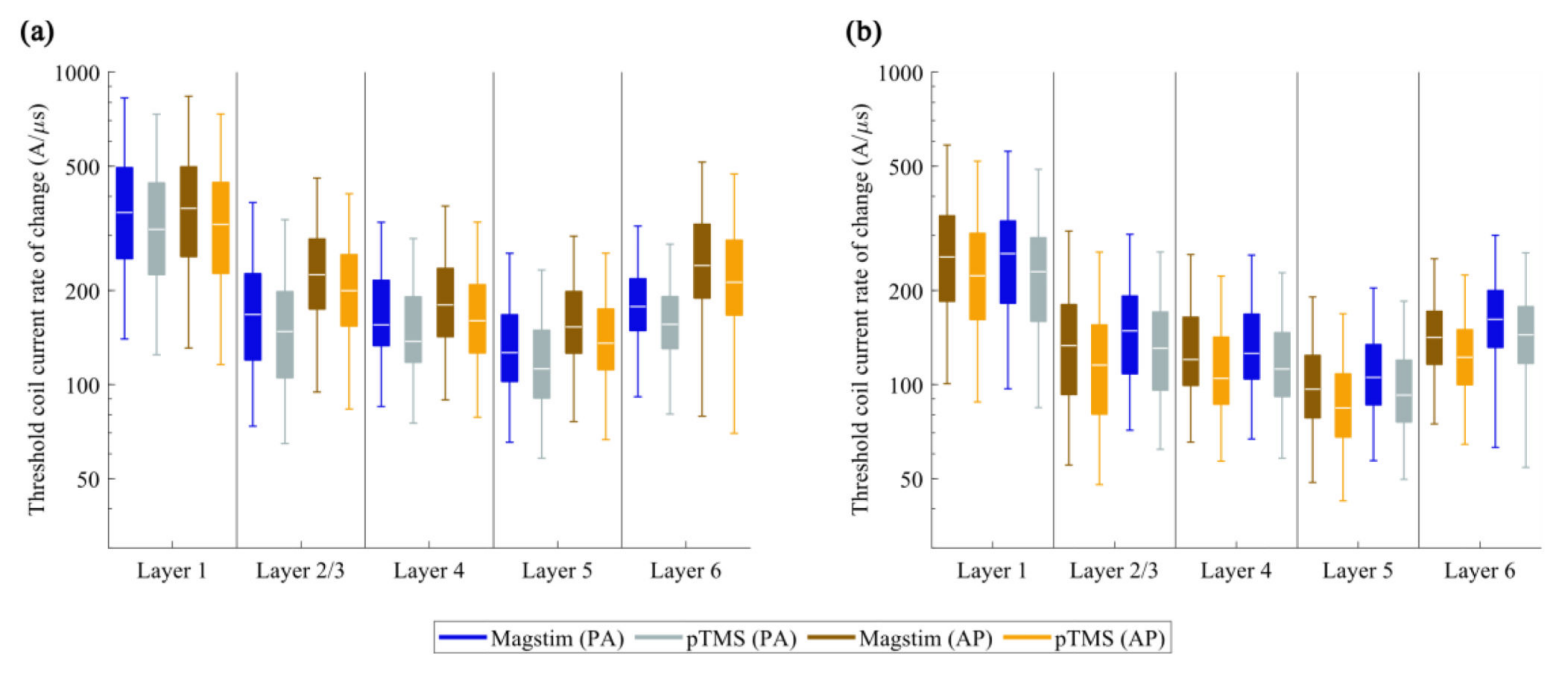
Comparison of modelled neural activation thresholds within the cortical area representing the hand muscle. The modelled thresholds for the conventional TMS devices and the programmable TMS are shown in log scale for **(a)** monophasic stimuli and for **(b)** biphasic stimuli for the posterior-anterior and anterior-posterior direction of the primary current in each layer. Each boxplot includes the data from five clones with the outliers removed.

Fig. 4 shows the activation thresholds of each layer within the cortical area approximating the hand muscle representation for each stimulus waveform and current direction. Each boxplot includes the data from the five neuron clones within the relevant layer. Overall, the activation thresholds for biphasic stimuli (Fig. 4 (b)) are lower than for monophasic stimuli (Fig. 4 (a)). Additionally, monophasic stimuli have lower thresholds when applied in the posterior-anterior current direction, while biphasic stimuli have lower thresholds when their initial current direction is in the anterior-posterior direction, due to their dominant second phase. This agrees with the results of previous computational and experimental studies of the motor threshold using the MagPro stimulators and coils [5, 10].

**Fig. 5:**
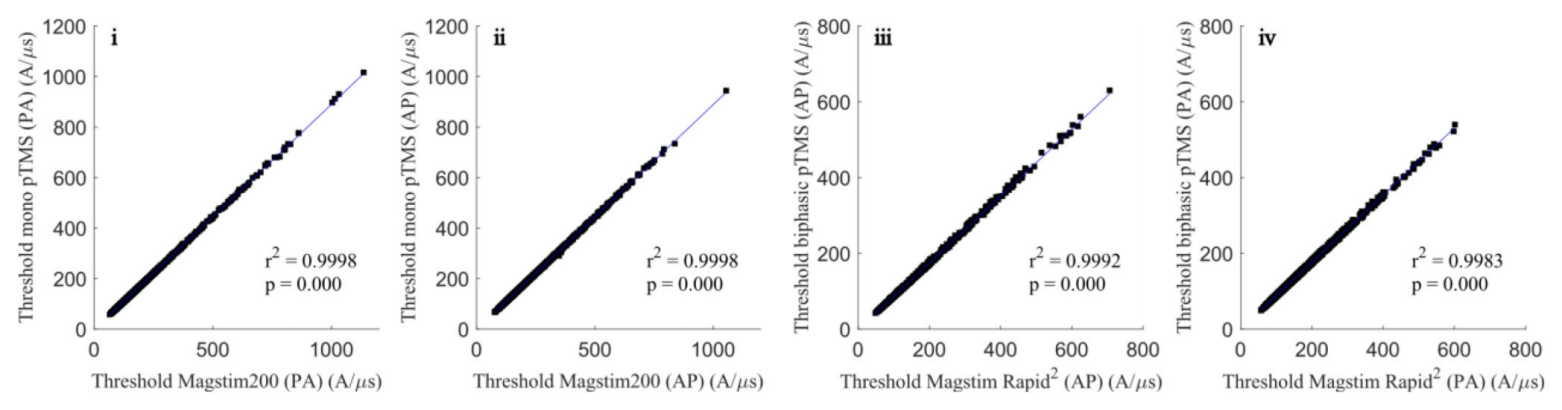
Correlation between the threshold coil current rate of change for Magstim pulses and their pulse-width modulated equivalents. The thresholds for the pTMS pulses are plotted against the thresholds for their Magstim references for **i**. the monophasic stimuli in the posterior-anterior direction, **ii**. the monophasic stimuli in the anterior-posterior direction, **iii**. the biphasic stimuli in the anterior-posterior direction and **iv**. the biphasic stimuli in the posterior-anterior direction. The linear regression is displayed in blue for each waveform and current direction.

As evident in Fig. 3 (c) and Fig. 4, the thresholds for the PWM pulses are consistently lower than for the Magstim pulses across the layers for all waveforms and current directions. To quantify the relationship between the thresholds for the different stimulation devices, Fig. 5 shows the threshold of each neuron in the hand muscle representation for Magstim pulses (abscissa) and pTMS pulses (ordinate) for monophasic (i-ii) and biphasic pulses (iii-iv). Linear regression revealed a strong correlation (r^2^ > 0.998, p = 0.000) between the thresholds of the two stimulation devices for all waveforms and current directions. The slopes of the lines of best fit were between 0.883 and 0.891. This analysis was repeated for the entire neuron population used in the model, which showed an equally strong correlation. This indicates that the PWM pulses approximate the neural activation by conventional pulses very closely. Additionally, the uniform difference observed in the activation thresholds and the slope of the linear regression lines suggest that the energy required to stimulate a desired neuron population is lower for the PWM pulses than for the Magstim pulses used here. Furthermore, the activation thresholds for different waveforms depend on the coil orientation but biphasic waveforms appear to be less sensitive to the orientation than monophasic waveforms.

## IV Limitations

In this study, stimulation pulses generated by resonance-based and PWM-based architectures are compared under ideal conditions. The head model, the stimulation coil, as well as all other parameters within the model are kept constant while the temporal waveform is varied. It should be noted that variables other than the shape of the waveform have a large impact on the neural response to stimulation. For instance, the type of stimulation coil heavily influences the spatial distribution of the induced electric field [11]. Even two coils of the same type but from different manufacturers may have different properties which must be considered when comparing the effects of different stimulus waveforms. In practice, any differences shown here between the threshold effects are likely to be obscured by the inherent variability of TMS effects. Apart from the stimulation parameters, additional factors such as the time awake and the circadian rhythm influence the effect of TMS [12] and common outcome measures such as the amplitude of motor evoked potentials show large variability from one trial to the next in the same subject [13]. Additionally, while this study only looks at one example brain from the SimNIBS database, the effects of TMS may vary between individuals. In future work, this analysis should be extended to head models of different brains and validated under practical conditions such as measuring the motor thresholds of human participants.

## Conclusion

In this work, morphological neural models integrated with transcranially induced electric fields are used to directly compare the neural response to pulse-width modulated TMS versus conventional TMS pulses. For both monophasic and biphasic stimulus waveforms, the effects of the different pulse types are shown to be highly correlated, which demonstrates that PWM pulses can approximate the conventional resonance-based pulses well. These results pave the way to explore new stimulation parameters and patterns using the pTMS architecture in the future.
